# Prevalence of Depression Among College-Goers in Mainland China: A Methodical Evaluation and Meta-Analysis

**DOI:** 10.1097/MD.0000000000002071

**Published:** 2015-12-18

**Authors:** Cai Xiao Jiang, Zhan Zhan Li, Peng Chen, Li Zhang Chen

**Affiliations:** From the Department of Epidemiology and Health Statistics, School of Public Health (CXJ, ZZL, LZC); and Xiangya Medical School, Central South University, Changsha, Hunan Province, China (PC).

## Abstract

There are no proper statistics available to assess how much of a burden it is to them. This study was conducted to gauge the pooled prevalence and offer evidence in support of few prevention and regulation strategies.

A methodical literature search was conducted with the help of the Web of Knowledge, PubMed, Chinese Web of Knowledge, Wanfang, and Chongqing VIP databases. Furthermore, articles published from 2000 to 2014, reporting about the estimated prevalence of depression among college students in mainland China, were covered as well. In this study, a meta-analysis was deployed to approximate the overall prevalence of depression among college-goers in mainland China.

A total of 45 studies were conducted on 50,826 participants. The average pooled prevalence of depression was 30.39% (26.38–34.55%). Subgroup analyses showed that 29.45% (22.88–36.48%) were men and 28.65% (23.44–34.16%) were women. Furthermore, 28.10% (22.83–33.70%) were from the northern part and 32.44% (26.67–38.48%) were from the southern. The prevalence of depression was 30.45% (23.96–37.36%) for sample sizes of <500 subjects, 30.99% (25.08–37.23%) for samples with 500 to 1000 people, and 29.54% (33.32–37.33%) in case of samples with >1000 people. Publications between 2000 and 2006 showed a depression prevalence of 8.45% (22.34–35.00%), whereas 30.52% (21.30–40.61%) and 31.79% (27.31–36.45%) were the corresponding values according to publications during 2007 to 2011 and 2012 to 2014.

The prevalence of depression among college students in mainland China had reached the world's epidemic level.

## INTRODUCTION

Symptoms of depression are quite prevalent. Severe mental illness is typically characterized by sad, nervous, hopeless, or anxious feelings.^1^ According to the World Health Organization,^2^ depression is now a health issue faced by people worldwide,^3^ particularly college students.^4,5^ A study by Ibrahim et al observed that the prevalence level of depression was 10 to 85%, with a weighted mean prevalence of 30.6% in case of university students all over the world.^6^ Depression prevalence is higher among college students compared to the general populace.^7^ This is because the time in university constitutes a special transitory period in terms of social interactions, changing of habitation, and financial circumstances.^8^ Also, during this period, the students have to make numerous vital decisions, which amplifies pressure on them. Symptoms of depression disturb the students’ tasks in day-to-day life, such as scholarly performance and social functioning.^9^ Additionally, the students’ activity and productivity may be devastated by depression.^3^

With the economic and cultural development in the modern-day China, an increasing number of people are getting the opportunities to join university. As a matter of fact, the current number of college students in mainland China is at an all-time high. Meanwhile, the competition has become stiffer and stiffer. Unfortunately, depression among college students too has become exceedingly prevalent and widespread.^10^ Without proper and prompt treatment, depression may lead to adverse effects in life. Numerous studies have reported that teenage depression heightens the depressive mood in adults,^11^ and extreme cases may even lead to suicide. It is, therefore, essential to study the status of depression among college-goers in mainland China, although there is a lack of a national survey to gauge the burden of depression on the said population. This study carried out a systematic review and meta-analysis of depression among college students in mainland China to approximate the pooled prevalence and come up with evidence in support of depression prevention and regulation strategies.

## METHODS

### Ethical Approval

The ethical approval was not necessary in the review articles.

### Literature Search

Written works on depression among students in mainland China were obtained by perusing through the PubMed, Wanfang, Web of Knowledge, Chinese Web of Knowledge, and Chongqing VIP databases. The literature was restricted to the Chinese and/or English language and articles published during the period between January 2000 and September 2014. The following are the keywords that were used during the search in all the databases: depression, depressive disorders, depressive symptoms, university students, prevalence, college students, undergraduate students, adolescents, and/or young adults. Two independent reviewers (PC and ZZL) went through all the identified titles and abstracts. The study exempted clinical trials.

### Criteria for Incorporation and Exclusion

In the methodical review and meta-analysis, the selected studies had to meet the following criteria for inclusion: (1) cross-sectional study; (2) the study samples had to be college-goers in mainland China; (3) the studies’ objectives were to assess the prevalence of depression; (4) depression had to be monitored using a self-rating depression scale (SDS).

Exclusion criteria were (1) utilizing other scales other than SDS to screen depression; (2) repetitive study and review; (3) insufficient reporting of results; (4) clinical trials.

### Extraction of Data

Data was extracted by 2 investigators who separately used a standardized data extraction form from the selected studies. In case of a dispute between the 2 investigators, a discussion was held to arrive at an agreement. Information obtained from each study included: author's first name, year of publication, region, type of study, age, gender ratio (male/female), size of sample, number of students suffering from depression, and prevalence.

### Statistical Analysis

The pooled prevalence of depression and subgroups were computed using StatsDirect. The models used were the fixed-effects model or random-effects model. The model to be used was determined by whether or not there existed heterogeneity among studies. If *I*^2^ exceeded 50%, the random model was used. Subgroup analyses were also conducted based on gender, geographical location, size of sample, and year of publication. Cochran's *Q* statistic and the *I*^2^ statistic were used to evaluate heterogeneity among studies. In the *Q* test, *P* < 0.10 indicated lack of heterogeneity among studies. *P* < 0.10 rather than 0.05 was deemed as significant heterogeneity for the χ^2^-based *Q* testing. A value of 0% for *I*^2^ indicated lack of heterogeneity, whereas an increasing percentage was an indicator of higher heterogeneity. In other words, *I*^2^ was used to estimate total variation across studies that were due to heterogeneity rather than chance (<25% was to be considered low heterogeneity, 25–50% as moderate, and > 50% as high-level heterogeneity). The funnel plot was used to examine potential publication bias. Funnel plot asymmetry was evaluated using Egger's linear regression test, where *P* < 0.05 was considered to be illustrative of statistically significant publication bias.

## RESULTS AND DISCUSSION

### Search Result

**FIGURE 1:**
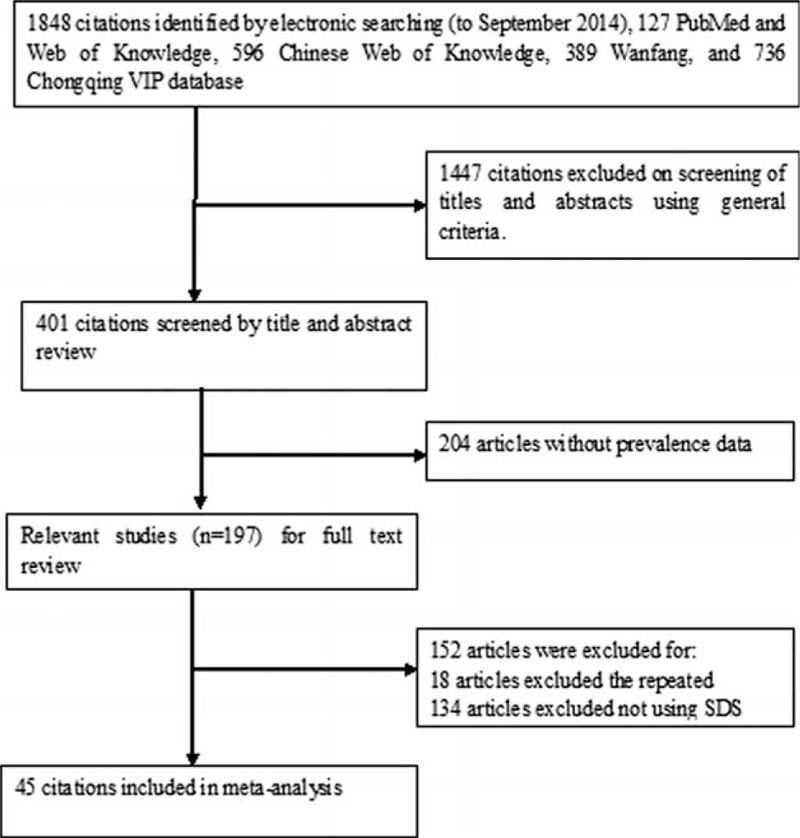
Flow diagram of included/excluded studies.

A thorough search of available databases yielded 1848 related articles, and after a careful examination of each article's titles and abstracts, a total of 197 articles were retrieved. Of the retrieved articles, an additional 152 articles were deemed ineligible after careful reading and were therefore excluded. Finally, 45 articles whose dates of publication were from January 2000 to September 2014 were deemed eligible and were thus included in the meta-analysis. Figure 1 showcases the flow diagram of the search process.

### Features of Included Studies

The reviewed articles documented population sizes ranging from 176 to 6009, with a total sample size of 50,826. Twenty-one of these articles documented results from Northern China (n = 14,968), whereas 24 articles documented results from Southern China (n = 35,858). Additionally, 26 of the included articles provided individual data for both men and women. Table 1 indicates the characteristics of each study on the prevalence of depression.

**TABLE 1:**
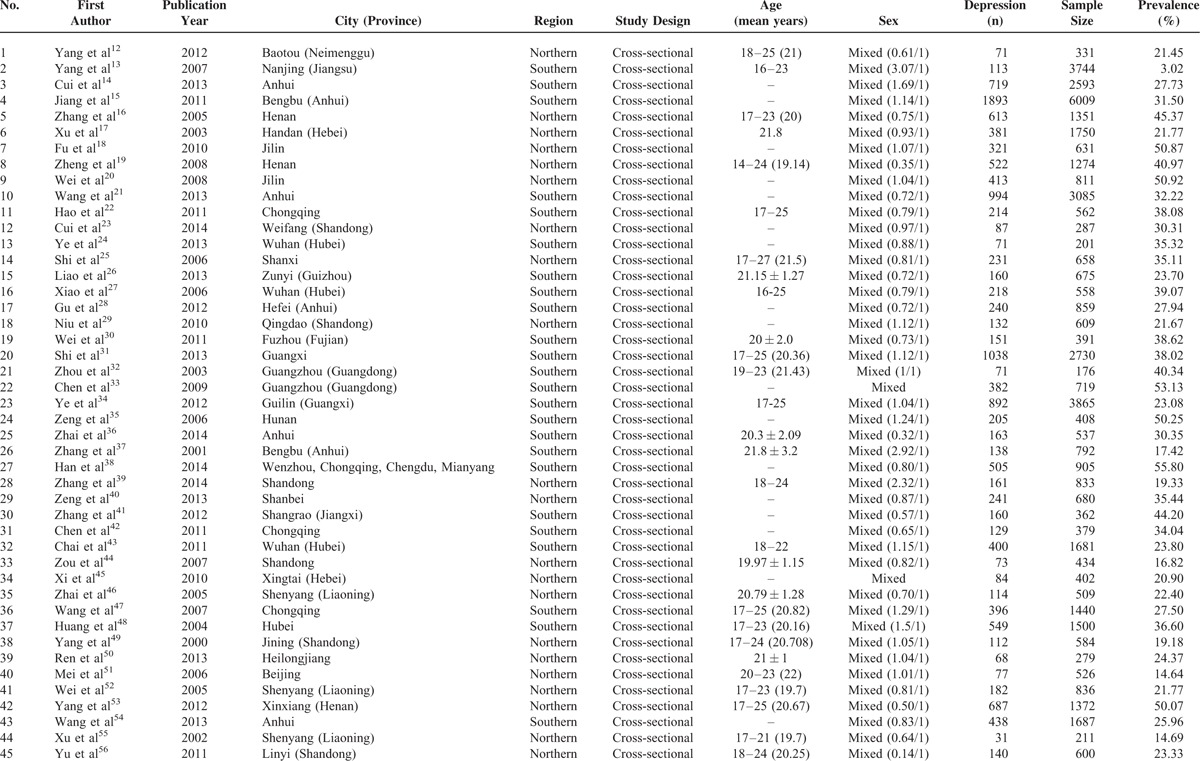
Characteristics of Studies on the Prevalence of Depression

### Prevalence of Depression

**FIGURE 2:**
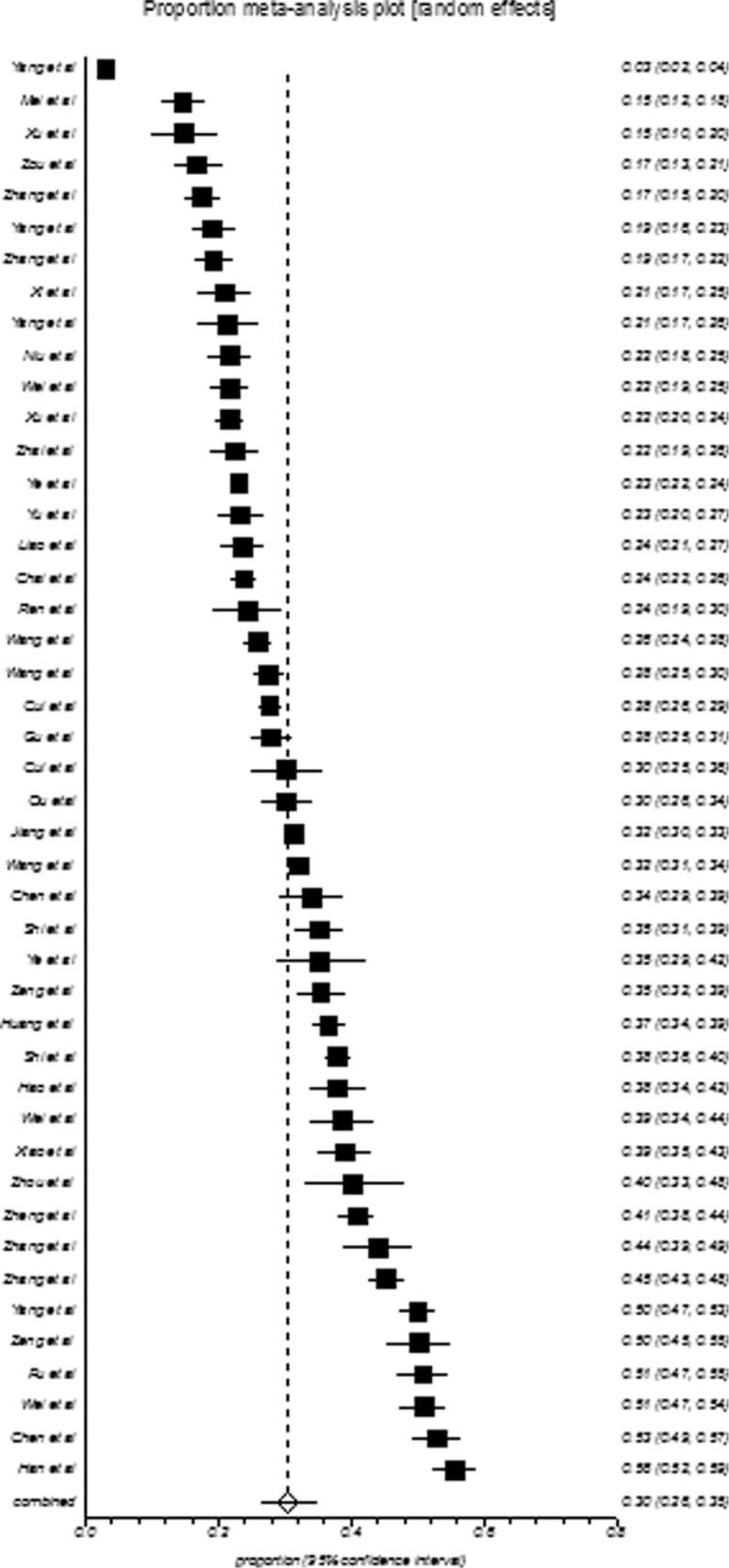
Forest plot of prevalence of depression for total college students.

**FIGURE 3:**
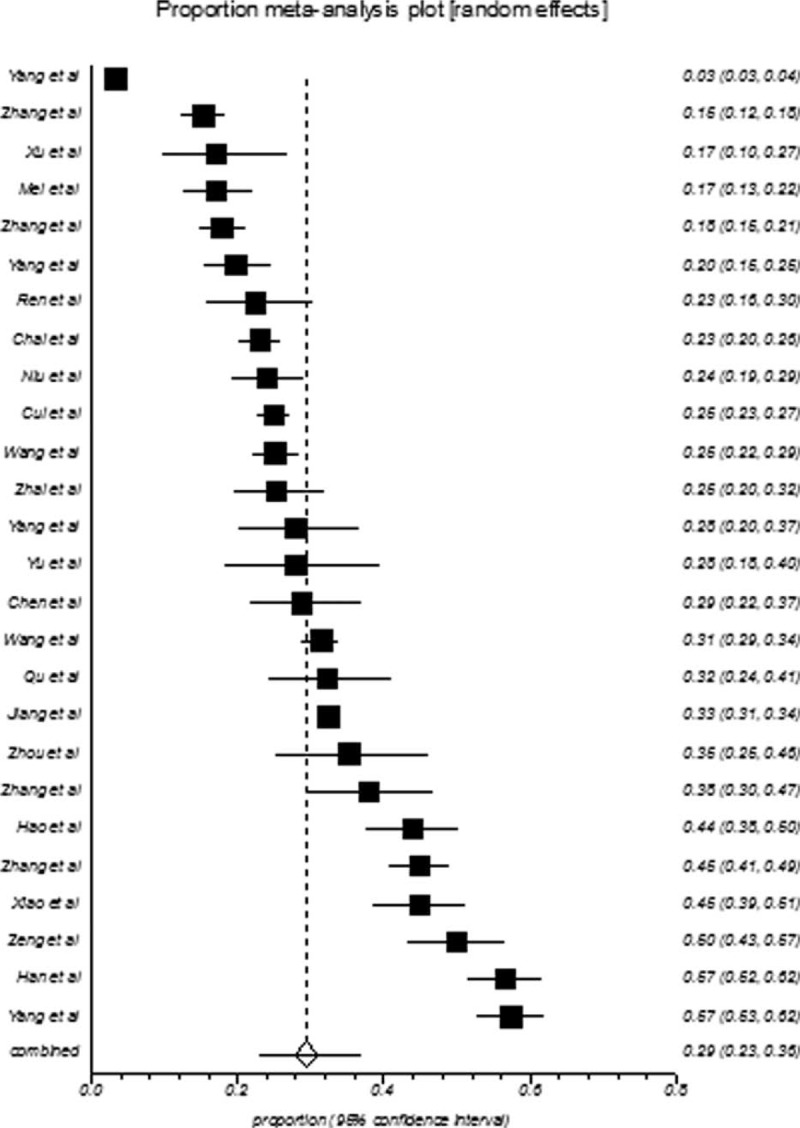
Forest plot of prevalence of depression for men.

**FIGURE 4:**
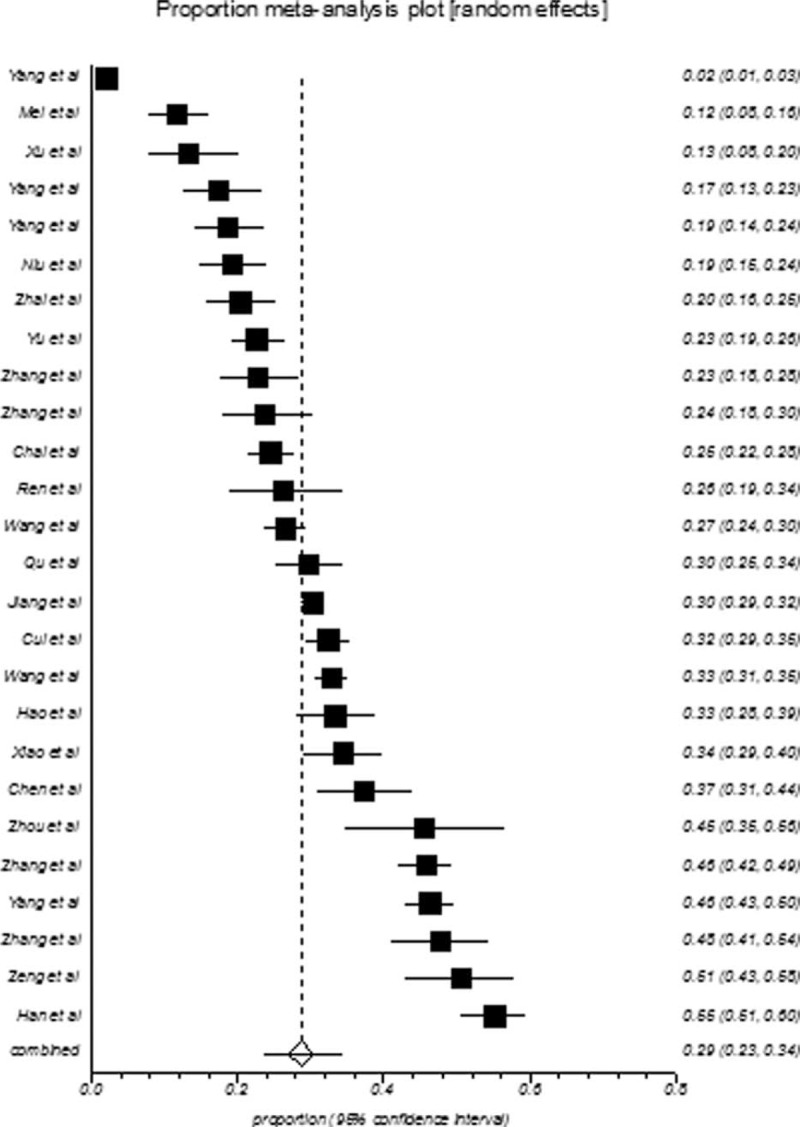
Forest plot of prevalence of depression for women.

College students in mainland China showed an overall depression prevalence value that ranged between 3.02% and 55.80% and an overall meta-analysis prevalence of 30.39% (95% CI: 26.38–34.55%, Fig. 2). The prevalence of depression in college-goers of mainland China also showed indication of high-level heterogeneity between the studies (*I*^2^ = 99%, *P* < 0.001). The population of college students is further subgrouped according to gender, geographical location, sample size, and publication year, and the pooled prevalence of these subgroups is presented in Table 2. The summarized prevalence of depression in men (29.45%, 95% CI: 22.88–36.48%, Fig. 3) was found to be higher (OR = 1.02, 95% CI: 0.95–1.10) than that of females (28.65%, 95% CI: 23.44–34.16%, Fig. 4). It was also apparent that the pooled prevalence estimate increased over time. For example, the pooled prevalence estimate was 28.45% (95% CI: 22.34–35.00%) from 2000 to 2006, increasing to 30.52% (95% CI: 21.30–40.61%) during 2007 to 2011, and further increasing to 31.79% (95% CI: 27.31–36.45%) during 2012 to 2014. Information regarding the heterogeneity of the data as well as the publication bias is depicted in Table 2.

**TABLE 2:**
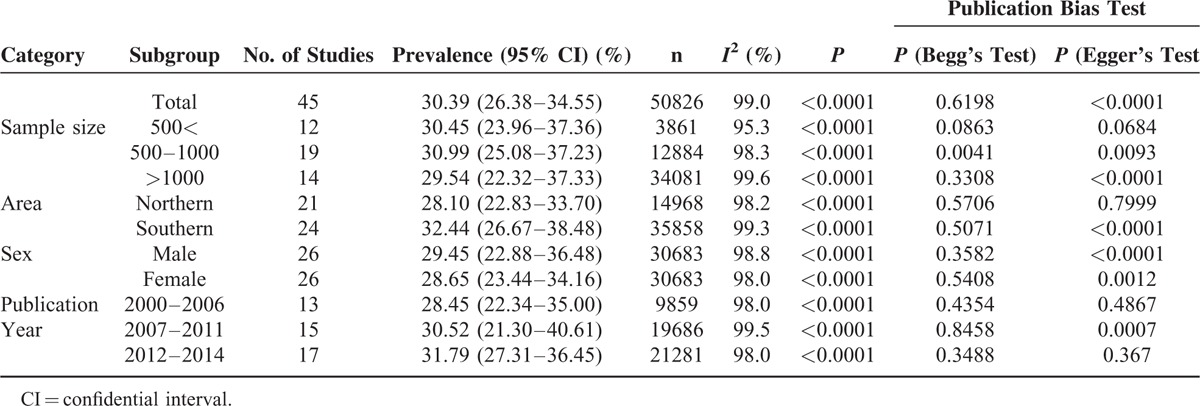
Prevalence of Depression According to Different Categories

### Publication Bias

Begg's test and Egger's test were used to give value to the publication bias. No significant bias in the included studies (*P* = 0.6198) was revealed using the modified Begg's linear regression test. However, a significant publication bias in the included studies was observed using Egger's test (*P* < 0.0001) (Table 2).

## DISCUSSION

A total of 45 studies covering >50,000 college students were included in this systematic study. The meta-analysis, conducted as part of this study, found that 30.39% (26.38–34.55%) of mainland Chinese college students suffered from depression. The rate is higher than the 22.8% incidence found in the older adult population of China.^57^ The study's observation of depressive symptoms was higher than the rate in the Chinese general population (7.2%).^58^ The results indicated the following: there are certain unique characteristics of college students, such as increased social interactions and changing residential and financial situations, which may increase the occurrence of depression.^8^ Furthermore, the students have to deal with a very difficult period of transition. This creates additional stress in both their future employment situations and lives in general. During this period, college students are forced to make key decisions that would increase the stress and pressure that is placed on them. Thus, it can be said that their student status may make them prone to depression.^6^

The prevalence of depression in college students over time has been noted to have increased by many studies.^59,60^ These findings are consistent with our results. The following reasons are cited to explain these trends. First, the cultural and economic development of the modern Chinese society has given more people the opportunity to get a university education. The rise in college enrollment expansion in China has created a serious problem with respect to the creation of adequate employment opportunities for Chinese college graduates. The surplus of underemployed college graduates means increased stress and pressure in finding adequate employment. Second, the major lifestyle and economic changes in China may impact interpersonal communication styles, further increasing pressure on college students. This could also be the result of increasing numbers of publications.

There are differences observed in the occurrence of depression between different countries as well as within the same country. A possible reason for these differences could be race. Steptoe et al found that there were highly significant differences in the prevalence of depression between countries. The highest levels were found in Korea, Taiwan, South Africa, Japan, and Thailand, and the lowest values in the Netherlands, Venezuela, and Belgium.^61^ The overall prevalence ranged between 3.02% and 55.80% in the systematic review. The overall meta-analysis prevalence was 30.39%. This was higher than in Korea,^62^ Turkey,^63,64^ US,^65^ Germany,^66^ and the UK,^67^ and lower than in India,^68^ Thailand,^69^ Poland,^66^ Iran,^4^ Ghana,^70^ and Bulgaria.^66^ The prevalence rates of depression found in a world-wide systematic review of university students ranged from a low of 10% to a high of 85% with a weighted mean prevalence of 30.6%. As these results are similar to our results,^6^ it is clear that the prevalence of depression among mainland China's college students was at the world's epidemic level. Also, as different appraisal standards and measurement tools were utilized in these studies, there could have been the difference in prevalence rates.^4^

Among college students, gender has been indicated to have a very important influence on the depression epidemic. Female students face greater degrees of depression,^61,71,72^ and the depression is more prevalent among women than men. Women have more tendencies to have moderate to serious depression (18.0% vs 9.0%)^73^ than men, according to Thomas L. Schwenk. The divergence may be due to social and cultural aspects. Another element that plays a significant role in the difference is biological conditions. In mainland China, male students have greater level of depression as researched by Tang et al. The following scenarios are the defined reasons for the difference: in old culture, the family and society have greater expectations from men; thus, men are more vulnerable to greater stress than women. Also, it might further intensify depression to overpower the depression mood^74^ because men are more prone to take the negative course. However, our investigation revealed that 29.45% of men and 28.65% of women experienced depression and we did not find any difference among gender. It does not have any effect on the development of depression on college students. Other past research too did not find difference due to gender.^6,67,75^ Sarokhani discovered that the prevalence of depression in a subgroup in Iran, which includes male and female students, was 28% (95% CI: 26–30%) and 23% (95% CI: 22–24%). Furthermore, no difference was found between the genders.^4^ In China, the idea of equality among men and women is becoming more and more accepted, which is the possible reason for the outcome of the study. Men and women tend to experience equal rights, and so the stress experienced by the college students may be the same.

The regional conflict might also affect the epidemiology of depression. Our research shows that the combined prevalence of depression in China's southern part was 32.44% (95% CI: 26.67–38.48%), whereas the combined prevalence in China's northern part was 28.10 (95% CI: 22.83–33.70%). The prevalence is more significant in the south than in the north. This analysis might be due to the difference in the economic status and medical conditions of the different regions. The southern region has a better economic status and medical condition compared to the north, which results in the depression being more distinguishable in the south. In other words, social, economic, and cultural environments are significant factors that influence students’ mental health.^10^

Only the outcome from the self-rating depression scale (SDS) was covered in this research. The SDS was developed in 1965 by Zung. It is a 20-item self-report assessment of depressive symptom severity that would reveal symptoms of depression and its severity entirety. The SDS is not be influenced by aspects such as age, gender, and economic situations, and is easy to complete. The scale is applicable to all sorts of professional and cultural traditions, typical age groups, or all types of mental patients. The SDS is a broadly used and a well-certified self-report instrument. We exclusively selected SDS in this meta-analysis to ensure that the measure of study was consolidated and the outcome was trustworthy.

There are some probable restrictions the meta-analysis may come across that might affect the outcome. First, the research that we did was limited to articles that were published in English and Chinese, and the unpublished studies that were not included may have caused an oversight. In other words, this action might have resulted in the exclusion of few articles about the prevalence of depression on college students. Second, as the study samples of the covered studies were not chosen in random, there might have been some preference while choosing and confounding, which could not be avoided. Third, this research involves publication bias. Lastly, extreme heterogeneity occurred in this research and when the study of the subgroup was done, heterogeneity still persisted. In addition, there was a dearth of essential data to search for the root of heterogeneity.

## CONCLUSIONS

To conclude, the prevalence of depression among college students in mainland China was assessed by meta-analysis, and the results indicate that the prevalence is at an epidemic level observed worldwide.
